# Joint modelling of multivariate longitudinal clinical laboratory safety outcomes, concomitant medication and clinical adverse events: application to artemisinin-based treatment during pregnancy clinical trial

**DOI:** 10.1186/s12874-021-01412-9

**Published:** 2021-10-09

**Authors:** Noel Patson, Mavuto Mukaka, Umberto D’Alessandro, Gertrude Chapotera, Victor Mwapasa, Don Mathanga, Lawrence Kazembe, Miriam K. Laufer, Tobias Chirwa

**Affiliations:** 1 School of Public Health, University of the Witwatersrand, Johannesburg, South Africa; 2 School of Public Health and Family Medicine, College of Medicine, University of Malawi, Blantyre, Malawi; 3 Mahidol Oxford Tropical Medicine Research Unit (MORU), Bangkok, Thailand; 4 Centre for Tropical Medicine, Nuffield Department of Medicine, University of Oxford, Oxford, UK; 5 Medical Research Council Unit, The Gambia at the London School of Hygiene and Tropical Medicine, Fajara, Gambia; 6 Department of Biostatistics, University of Namibia, Windhoek, Namibia; 7 Center for Vaccine Development and Global Health, University of Maryland, School of Medicine, Baltimore, MD USA

**Keywords:** Adverse events, Randomised controlled trials, Joint model, Drug safety, Concomitant medication

## Abstract

**Background:**

In drug trials, clinical adverse events (AEs), concomitant medication and laboratory safety outcomes are repeatedly collected to support drug safety evidence. Despite the potential correlation of these outcomes, they are typically analysed separately, potentially leading to misinformation and inefficient estimates due to partial assessment of safety data. Using joint modelling, we investigated whether clinical AEs vary by treatment and how laboratory outcomes (alanine amino-transferase, total bilirubin) and concomitant medication are associated with clinical AEs over time following artemisinin-based antimalarial therapy.

**Methods:**

We used data from a trial of artemisinin-based treatments for malaria during pregnancy that randomized 870 women to receive artemether–lumefantrine (AL), amodiaquine–artesunate (ASAQ) and dihydroartemisinin–piperaquine (DHAPQ). We fitted a joint model containing four sub-models from four outcomes: longitudinal sub-model for alanine aminotransferase, longitudinal sub-model for total bilirubin, Poisson sub-model for concomitant medication and Poisson sub-model for clinical AEs. Since the clinical AEs was our primary outcome, the longitudinal sub-models and concomitant medication sub-model were linked to the clinical AEs sub-model via current value and random effects association structures respectively. We fitted a conventional Poisson model for clinical AEs to assess if the effect of treatment on clinical AEs (i.e. incidence rate ratio (IRR)) estimates differed between the conventional Poisson and the joint models, where AL was reference treatment.

**Results:**

Out of the 870 women, 564 (65%) experienced at least one AE. Using joint model, AEs were associated with the concomitant medication (log IRR 1.7487; 95% CI: 1.5471, 1.9503; *p* < 0.001) but not the total bilirubin (log IRR: -0.0288; 95% CI: − 0.5045, 0.4469; *p* = 0.906) and alanine aminotransferase (log IRR: 0.1153; 95% CI: − 0.0889, 0.3194; *p* = 0.269). The Poisson model underestimated the effects of treatment on AE incidence such that log IRR for ASAQ was 0.2118 (95% CI: 0.0082, 0.4154; *p* = 0.041) for joint model compared to 0.1838 (95% CI: 0.0574, 0.3102; *p* = 0.004) for Poisson model.

**Conclusion:**

We demonstrated that although the AEs did not vary across the treatments, the joint model yielded efficient AE incidence estimates compared to the Poisson model. The joint model showed a positive relationship between the AEs and concomitant medication but not with laboratory outcomes.

**Trial registration:**

ClinicalTrials.gov: NCT00852423

## Background

There has been limited analysis of safety data being conducted in drug clinical trials compared to efficacy data. During drug clinical trials, scheduled and non-scheduled visits are conducted where multiple safety outcomes are collected including longitudinal clinical laboratory outcomes and clinical AEs [1, 2]. Data on concomitant medications defined as any medication/supplement taken by the trial participant other than the investigational product, are also repeatedly collected in order to support drug safety evidence. The repeatedly measured clinical laboratory outcomes are considered as objective and proxy indicators for drug-induced human body organ dysfunction and tend to be strongly associated with incidence of clinical AEs. For example, classic liver function biomarkers such as total bilirubin and alanine aminotransferase are usually used in assessment of drug safety [3]. However, each of the clinical laboratory safety outcomes is usually analysed separately based on summary statistics such as means or proportion of patients with elevated values for the safety markers [4, 5]. The clinical AE data is also usually analysed separately using the descriptive non-parametric methods (e.g. cumulative incidence function and crude incidence rate) [6–8]. Although ignored in most safety analyses, concomitant medication taken during the trial period can also influence the magnitude of drug safety estimates since the concomitant medication can interact with the treatment under investigation. Concomitant medication is usually also analysed separately (e.g. using mean cumulative function) [9]. The standard approaches that analysed each of the clinical laboratory safety data, clinical AEs and concomitant medication separately are inefficient and may yield biased estimates of AE incidence rate (i.e. clinical AEs occurrence over follow-up time) since they ignore the correlation structure of the multiple outcomes over the follow-up time, leading to information loss. Furthermore, the separate analyses present challenges in interpreting overall drug safety profile. Integrated approach in analysis of the clinical laboratory safety data, concomitant medication and clinical AEs through the use of joint models, can offer an opportunity to efficiently harness the available information towards enriching the drug safety estimates. The joint models can efficiently quantify how the incidence rate of AEs is associated with the multivariate longitudinal process of the multiple continuous laboratory-based measurements, accounting for baseline covariates, concomitant medications and unobserved heterogeneity that is not captured in the conventional analytical approaches of safety data, all of which can potentially confound the estimates.

Recently, there have been increased proposals for improved analysis of multivariate clinical laboratory data, concomitant medications and clinical AEs. Schildrout et al. [10] and Rosenkranz [11] discuss the utility of longitudinal modelling of clinical laboratory data using estimating equations and maximum likelihood. However, such separate longitudinal models ignore correlation between the multiple safety outcomes. Southworth and Heffernan proposed joint modelling approach that captures the joint behaviour of clinical laboratory safety data elevated values indicative of potential liver damage [12]. However, this was limited as the model did not use all the available information due to focus on extreme values and, further, such modelling is mainly suited for large sample sizes. Merz et al. [3] also recommended accounting for both multivariate longitudinal clinical laboratory data and clinical AEs, although they do not explicitly discuss how this can be practically achieved. Barker [9] demonstrates how mean cumulative function can be used as an exploratory tool in analysis number of concomitant medications taken over the follow-up time in order to support drug safety evidence interpretation. Building on these recent developments, this paper explores whether jointly modelling multivariate longitudinal clinical laboratory, concomitant medication and clinical AEs adjusted for baseline characteristics can be efficient in estimating AE incidence rate and useful in understanding joint evolution of the potentially correlated multiple safety outcomes.

In clinical trials with frequent AE occurrence (e.g. antimalarial treatment in pregnancy trials), event count models such as Poisson or negative binomial regression are considered important in quantifying the AE rate [13–16]. However, the AEs are also highly correlated with the cumulative concomitant medications and clinical laboratory safety outcomes. Joint modelling can offer an opportunity to explicitly model the correlations and account for the follow-up time, ensuring efficient and unbiased estimates. Despite the advanced development and evident benefits of joint modelling of multivariate longitudinal data [17–19], joint modelling has received limited attention in drug safety assessment. Recent statistical software developments have provided an opportunity for extending joint models to more different types of outcomes [20, 21], beyond the traditionally implemented joint models with single continuous longitudinal data and single survival outcome. In this paper, we present a joint model for longitudinal continuous clinical laboratory safety data (alanine amino transferase and total bilirubin), individual clinical AEs and concomitant medication. We focus on demonstrating the utility of the proposed model in assessing the association between the clinical AEs in relation to these outcomes controlling for baseline covariates. Furthermore, we assessed whether the joint model improved the estimates in log incidence rate ratio (IRR) on effect of treatment on clinical AEs compared to the conventional Poisson model. The proposed joint model is useful in providing evidence-based decisions in determining the most important clinical safety measurements to focus on for drug safety assessment especially in resource-constrained settings.

## Methods

### Data

Our proposed joint model was applied to data from PREGACT trial as the motivating data. PREGACT trial (ClinicalTrials.gov number, NCT00852423) was a multicentre, open-label randomized trial carried out in 4 African countries (Burkina Faso, Ghana, Malawi, and Zambia). The current work uses the data from Malawi. In Malawi, the trial was implemented between June 2010 and August 2013 and enrolled 870 pregnant women during their second or third trimester with falciparum malaria. The women were randomly allocated in a 1:1:1 ratio to be treated with artemether–lumefantrine (AL), amodiaquine–artesunate (ASAQ) or dihydroartemisinin–diperaquine (DHAPQ). The primary outcome was polymerase-chain reaction (PCR) adjusted cure rates at day 63.

The safety outcomes collected at baseline and during follow-up were AEs, total bilirubin, alanine aminotransferase, white blood cell and red blood cell counts. The patients were directly observed on days 0-2 (after receiving the dose). The patients were then asked to return to the clinic for follow-up visits on days 3 and 7 and then weekly thereafter until day 63. For this analysis, we focus on four outcomes; two clinical laboratory safety biomarkers (total bilirubin and alanine aminotransferase), clinical AEs and concomitant medication. The total bilirubin and alanine aminotransferase data was collected at enrolment, day 7, 14, 28 and 63. We focused on bilirubin and alanine aminotransferase biochemical parameters since they are key biochemical parameters in antimalarial drug safety assessment during pregnancy and were also measured in the PREGACT trial.

The clinical AE count, defined as cumulate number of AEs experienced by the end of follow-up time is the primary outcome of interest in the current analysis. In all the subsequent discussions, the clinical AEs that were defined as *definitely not related* (by the study physician) to the antimalarial drug treatment are not considered in developing the joint model to avoid spurious results. The concomitant medication outcome is also defined as cumulative number of reported concomitant medication use by the end of the follow up time for each patient. In the current study, we considered the reported concomitant medication use regardless of its intended use.

### Ethical considerations

The PREGACT trial was conducted in accordance with the Declaration of Helsinki and Good Clinical Practice guidelines. The trial obtained ethical clearance from ethics committee at the Antwerp University Hospital in Belgium and College of Medicine Research Ethics Committee at the University of Malawi [22]. Prior to enrolment, informed consent was also sought from the mother. Ethical approval was also obtained from University of the Witwatersrand Human Research Ethics Committee, prior to access and utilization of the data for the current analysis.

### Notation and joint model specification

Let each patient who was randomized and received at least a dose be denoted as *i* = 1, … …, *n*. Let ${V}_i=\left({v}_{i1}^{\mathrm{T}},\kern0.5em {v}_{i2}^{\mathrm{T}}\right)$ be a bivariate continuous clinical laboratory outcome vector. Specifically, in our context we consider a bivariate scenario; k = 1 is alanine amino transferase and k = 2 is total bilirubin. Each of the two continuous outcome vectors (*v*_*i*1_,  *v*_*i*2_) are of (*n*_*ik*_x1) dimension for the observed longitudinal measurements of the k-th outcome; *v*_*ik*_ = (*v*_*i*1*k*_, …….,  *v*_*ink*_)^T^. We accommodate the situations where observation times, *t*_*ijk*_ may differ between individuals and outcomes (*j* = 1,  … . ., *n*_*ik*_). For each patient, we let ${C}_i=\left({c}_{i1}^{\mathrm{T}},\kern0.5em {c}_{i2}^{\mathrm{T}}\right)$ represent a bivariate count outcome vector where k = 1 is for total number of AEs experienced during their follow-up time *T*_*i*_ and k = 2 for total number concomitant medication used over the follow-up time *T*_*i*_. A set of covariates that were collected at baseline for each individual are defined as ***X***_***i***_ = {*X*_1*i*_, *X*_2*i*_, … . ., *X*_*pi*_}.

### Conventional Poisson model for clinical AEs

In drug trials, where multiple safety outcomes are repeatedly collected to support safety evidence, clinical AEs are usually of primary interest. The occurrence of the clinical AEs is typically correlated with other safety-related outcomes such as alanine aminotransferase, total bilirubin and concomitant medication. Modelling these outcomes separately is inefficient since it insufficiently accounts for the correlations. Traditionally, modelling of clinical AEs count is done using the conventional Poisson model, where the Poisson mean response (of clinical AEs in this context), *φ*, is assumed to be equal to the variance Since the clinical AEs count is non-negative the logarithm of *φ* can naturally be linked with the baseline variables such that the conventional Poisson model for the clinical AE count can be presented as;

1 $$\log \left(\varphi \right)={\boldsymbol{X}}_{ik}^{\mathrm{T}}\boldsymbol{\beta}$$

Where *φ* represents Poisson mean response and ***β*** is a vector of coefficients (i.e. log IRR) for the corresponding vector of baseline covariates, ${\boldsymbol{X}}_{ik}^{\mathrm{T}}$, that included maternal age, gravidity, trimester at enrolment and treatment arm.. The model M1 has log link of the mean response of clinical AEs count and assumes a Poisson distribution of the clinical AEs. The coefficients of the covariates are interpreted on logarithm scale as expected change in the log of mean clinical AEs count per unit change in the covariate. Exponentiating the coefficients (i.e. log IRR) yields IRR. However, the estimated effect of treatment on clinical AEs from model M1 may be insufficient since it does not account for other factors that can confound the estimates e.g. concomitant medication and clinical laboratory outcomes. Alternatively, one can consider incorporating this additional information (of concomitant medication and clinical laboratory outcomes) as part of covariates in model M1. This can yield model M2 below.

2 $$\log \left(\varphi \right)={\boldsymbol{X}}_{ik}^{\mathrm{T}}\left(\boldsymbol{t}\right)\boldsymbol{\beta}$$

Since the concomitant medication and clinical laboratory outcomes (alanine aminotransferase, total bilirubin) are added in the model as time-varying covariates (with time *t*0, representing baseline covariates in M1), the vector of covariates adjusted in model M2 is ${\boldsymbol{X}}_{ik}^{\mathrm{T}}\left(\boldsymbol{t}\right)$ denoting the vector of covariates value at time t. However, both model M1 and M2 do not account for correlation overtime across the longitudinal alanine aminotransferase, longitudinal total bilirubin, concomitant medication and clinical AEs outcomes. In this context of multiple outcomes that are correlated overtime, joint modelling offers an opportunity to obtain improved and efficient estimates since it can efficiently account for the potential correlations and baseline covariates. The joint model also enables formal quantification of the relationship between correlated outcomes by estimating the strength of the association between the outcomes.

Most previously proposed joint models consist of two sub-models; longitudinal sub-model and time-to-event sub-model (where the time to event outcome is of primary interest). Here, we propose a joint model where the primary outcome of interest is count outcome (i.e. clinical AEs count) such that time to event outcome sub-model is replaced with count outcome sub-model. Since we assumed Poisson distribution of the clinical AEs count, the count outcome sub-model was specified as Poisson-distributed. Therefore, the joint model presented in this paper consist of two longitudinal continuous laboratory safety biomarkers sub-models (one for total bilirubin and one for alanine aminotransferase) two Poisson sub-models (one for the individual clinical AEs count and one for the individual concomitant medication count). The connecting of these outcomes sub-models is very flexible such that we could use random effects or expected value of outcomes [23] as detailed in the model specification below.

### Joint model formulation

As highlighted above, the joint model is formulated in such a way that clinical AE count is a primary outcome. In this section we describe the structures of the sub-models that made the joint model considered in this work. The estimates in all the models are considered on a logarithm scale.

#### Longitudinal sub-model

We modelled two continuous longitudinal clinical laboratory safety outcomes (i.e. total bilirubin and alanine aminotransferase) using a longitudinal sub-model based on a flexible linear mixed effects model that could accommodate nonlinear changes of the laboratory safety outcomes. The flexibility was achieved through the use of restricted cubic splines. Each continuous longitudinal clinical laboratory outcome *v*_*i*1_, *v*_*i*2_ was assumed to be normally distributed with mean μ_*k*_ and variance ${\delta}_k^2$ (k = 1, 2) on a logarithm scale. Each continuous longitudinal clinical laboratory outcome is modelled using the sub-model below

3 $${v}_{ik}(t)={\boldsymbol{X}}_{ik}^{\mathrm{T}}(t)\boldsymbol{\beta} +{\boldsymbol{Z}}_{\boldsymbol{i}}(t){\boldsymbol{b}}_{\boldsymbol{i}}+{\varepsilon}_{ik}(t),i=1,\kern0.5em \dots \kern0.5em \dots, n;\kern0.5em \mathrm{k}\kern0.5em =\kern0.5em 1,\kern0.5em 2$$

where *ε*_*ik*_(*t*) is the error term observed at time t for the kth outcome from patient i. The error terms for the model are assumed to be independent, identical and normally distributed with mean 0. The mean response model ${\boldsymbol{X}}_{ik}^{\mathrm{T}}(t)\boldsymbol{\beta}$, specified as a linear function of the covariates at a given time for the outcome k is flexible such that it can accommodate time-varying covariates. The ***Z***_***i***_(***t***) is an indicator vector of random effects for kth outcome for patient *i* at time t such that it takes the value of 1 when there is a random effect and 0 otherwise. The vector of the patient-specific shared random effects ***b***_***i***_ is assumed to have a multivariate normal distribution with mean 0 and variance-covariance matrix **Σ**, s.t. (***b***_***i***_) **∼** ***MVN***(**0**, ***∑***).

##### Alanine aminotransferase sub-model

Based on M1, considering patient-specific random intercept as our shared parameter of interest, linking the outcomes, the longitudinal sub-model for alanine aminotransferase can be written simply as M4 below;

4 $${v}_{i1}(t)={\boldsymbol{X}}_{i1}^{\mathrm{T}}(t)\boldsymbol{\beta} +{\boldsymbol{b}}_{\mathbf{0}\boldsymbol{i}\mathbf{1}}$$

where *b*_0*i*1_ is the patient specific random intercept corresponding to the alanine aminotransferase outcome.

##### Total bilirubin sub-model

Similarly, the longitudinal sub-model for total bilirubin, M5, can be formulated as;

5 $${v}_{i2}(t)={\boldsymbol{X}}_{i2}^{\mathrm{T}}(t)\boldsymbol{\beta} +{\boldsymbol{b}}_{\mathbf{0}\boldsymbol{i}\mathbf{2}}$$

where *b*_0*i*2_ is the patient specific random intercept corresponding to total bilirubin outcome.

#### Count sub-models

We considered two event count sub-models; one for concomitant medication count and the other for the AE count. The two count sub-models were derived from a modified form of the generic Poisson model. The count sub-model was as follows;

##### Concomitant medication count sub-model

Assuming that the concomitant medication count had a Poisson distribution, its sub-model was defined as

6 $$\log \left(\varphi |{\boldsymbol{b}}_{\mathbf{03}\boldsymbol{i}}\right)={\boldsymbol{X}}_{ik}^{\mathrm{T}}(t)\boldsymbol{\beta} +{\boldsymbol{b}}_{\mathbf{03}\boldsymbol{i}}{\boldsymbol{\alpha}}_{\mathbf{3}}$$

such that log(*φ*| ***b***_**03*i***_) represents the logarithm of the Poisson mean response, *φ*, conditional on the patient-specific random intercept, ***b***_**03*i***_ for concomitant medication count (i.e. the concomitant medication count sub-model is linked with the AE count sub-model in the joint model via the random intercept). The ${\boldsymbol{X}}_{ik}^{\mathrm{T}}(t)\boldsymbol{\beta}$ is the linear predictor for the Poisson model has a logarithm link function and contains a vector of fixed effects ***β*** corresponding to the baseline covariates. The *α*_3_ represents log incidence rate ratio per unit increase in the patient-specific deviation from the mean random intercept of the reported concomitant medication use.

##### Clinical AE count sub-model

Considering that AE count was the primary outcome of interest and assuming a Poisson distribution for the AE count, the AE count sub-model was formulated as;

7 $$\log \left(\varphi |{\boldsymbol{b}}_{\mathbf{01}\boldsymbol{i}},{\boldsymbol{b}}_{\mathbf{02}\boldsymbol{i}},{\boldsymbol{b}}_{\mathbf{03}\boldsymbol{i}}\right)={\boldsymbol{X}}_{ik}^{\mathrm{T}}(t)\boldsymbol{\beta} +{\boldsymbol{b}}_{\mathbf{01}\boldsymbol{i}}{\boldsymbol{\alpha}}_{\mathbf{1}}+{\boldsymbol{b}}_{\mathbf{02}\boldsymbol{i}}{\boldsymbol{\alpha}}_{\mathbf{2}}+{\boldsymbol{b}}_{\mathbf{03}\boldsymbol{i}}{\boldsymbol{\alpha}}_{\mathbf{3}}$$

The log(*φ*| ***b***_**01*i***_, ***b***_**02*i***_, ***b***_**03***i*_) represents the logarithm of Poisson mean response, *φ*, conditional on the shared random intercepts for the two longitudinal clinical laboratory outcomes and the concomitant medication count. Each random intercept for the longitudinal sub-models and concomitant medication sub-model (*b*_01*i*_, *b*_02*i*_, *b*_03*i*_) is linked to the linear predictor ${\boldsymbol{X}}_{ik}^{\mathrm{T}}(t)\boldsymbol{\beta}$ for the Poisson model. This yields random effects association structure where *α*_1_, *α*_2_, *α*_3_ estimate the strength of the association between the respective continuous longitudinal clinical laboratory outcome, concomitant medication count and the AE count; the *α*_1_, *α*_2_, *α*_3_ represent log incidence rate per unit increase in the patient-specific deviation from the mean random intercept of a respective continuous longitudinal clinical laboratory outcome or the concomitant medication count outcome. For example, *α*_1_ represents log incidence rate ratio per unit increase in the patient-specific deviation from the mean random intercept of a respective log alanine aminotransferase.

Alternative clinically meaningful formulation of the AE count sub-model could be linked to the expected value of the respective continuous longitudinal clinical laboratory outcome with the linear predictor of the Poisson model. This yields the current value association structure where the *α*_1_, *α*_2_, *α*_3_ represent log incidence rate ratio per unit increase of the respective continuous longitudinal clinical laboratory outcome, at time t. Current value association structure is very important and clinically plausible when linking continuous outcomes. In our cases, given the two continuous longitudinal clinical laboratory outcomes (i.e. log alanine aminotransferase and log total bilirubin) ***v***_**1**_, ***v***_**2**_, under the current value association structure, we can modify the AE count sub-model as;

8 $$\log \left(\varphi \right)={\boldsymbol{X}}_{ik}^{\mathrm{T}}(t)\boldsymbol{\beta} +\boldsymbol{E}\left[{\boldsymbol{v}}_{\mathbf{1}}\left(\boldsymbol{t}\right)|{\upmu}_{\mathbf{1}}\left(\boldsymbol{t}\right)\right]{\boldsymbol{\alpha}}_{\mathbf{1}}+\boldsymbol{E}\left[{\boldsymbol{v}}_{\mathbf{2}}\left(\boldsymbol{t}\right)|{\upmu}_{\mathbf{2}}\left(\boldsymbol{t}\right)\right]{\boldsymbol{\alpha}}_{\mathbf{2}}+{\boldsymbol{b}}_{\mathbf{03}\boldsymbol{i}}{\boldsymbol{\alpha}}_{\mathbf{3}}$$

The ***E***[***v***_**1**_(***t***)| μ_**1**_(***t***)] and ***E***[***v***_**2**_(***t***)| μ_**2**_(***t***)]***α***_**2**_ are the expected current values of log alanine aminotransferase and total bilirubin respectively. It can be noted from M6 that the concomitant medication count, ***v***_**3**_, sub-model is still linked to the AE count sub-model via a random intercept ***b***_**03*i***_. We maintained the random intercept (*b*_03*i*_*α*_3_) parameterization since this efficiently deals with any potential over-dispersion problem encountered in Poisson models. Therefore, model M8 contains a mixture of both the expected current value and random effects association structures. Since both continuous outcomes (the log alanine aminotransferase and log total bilirubin) are assumed to be normally distributed (i.e. with identity link), the model can further be simplified as;

9 $$\log \left(\varphi \right)={\boldsymbol{X}}_{ik}^{\mathrm{T}}(t)\boldsymbol{\beta} +\left[{\upmu}_{\mathbf{1}}\left(\boldsymbol{t}\right)\right]{\boldsymbol{\alpha}}_{\mathbf{1}}+\left[{\upmu}_{\mathbf{2}}\left(\boldsymbol{t}\right)\right]{\boldsymbol{\alpha}}_{\mathbf{2}}+{\boldsymbol{b}}_{\mathbf{03}\boldsymbol{i}}{\boldsymbol{\alpha}}_{\mathbf{3}}$$

In this paper, we focus on reporting results from the M9 association structure since it is more clinically meaningful since it has association parameters that are directly interpretable in clinical practice.

#### Joint model likelihood and estimation

The estimates for the joint model are obtained by maximizing the marginal likelihood of the joint distribution of the observed data and the random effects [23]. The likelihood function for the observed data can be specified as;

10 $${L}_i\left(\theta \right)={\prod}_{i=1}^nf\left({\boldsymbol{V}}_{\boldsymbol{i}}|{\boldsymbol{b}}_{\boldsymbol{i}},\boldsymbol{\theta} \right)f\left({\boldsymbol{T}}_{\boldsymbol{i}},{\boldsymbol{C}}_{\boldsymbol{i}}|{\boldsymbol{b}}_{\boldsymbol{i}},\boldsymbol{\theta} \right)f\left({\boldsymbol{b}}_{\boldsymbol{i}}|\boldsymbol{\theta} \right)d{\boldsymbol{b}}_{\boldsymbol{i}}$$

where ***θ*** represents the set of parameters of interest to be estimated including both the fixed and random effects (e.g. association parameters ***α***_**1**_, ***α***_**2**_, ***α***_**3**_, and coefficients for the baseline covariates, **β**). Component *f*(***b***_***i***_| ***θ***) is a normal density function for the random effects conditional on ***θ*** and *f*(***V***_***i***_| ***θ***) is a normal density function for the log total bilirubin and log alanine aminotransferase conditional on ***θ*** and ***b***_***i***_. The *f*(*T*_*i*_, ***C***_***i***_| ***b***_***i***_, ***θ***) represents Poisson density function conditional on ***θ*** and ***b***_***i***_; patient-specific total follow up days is *T*_*i*_ and ***C***_***i***_ represents total events recorded (for AE count sub-model and concomitant count sub-model). Since obtaining the log likelihood requires integrating out the random effects, it becomes challenging as number of random effects increases such that the traditionally used adaptive Gauss-Hermite quadrature would be limited (to handle the large number of random effects in our complex joint model). To mitigate this problem, we employed Monte Carlo integration technique since the number of draws it makes from the random effects do not need to change with increase in number of random effects [23, 24] (hence reducing the computation burden). Estimation of our models was done using merlin in Stata [21]. Only 7 patients had missing data points and the data missing mechanism was considered completely at random.

### Statistical data analysis

Firstly, we computed the summary of the baseline characteristics for the women at enrolment. We presented means with respective standard deviation for the continuous variables (e.g. maternal age, gestational age) and frequencies with respective percentages for the categorical variables (e.g. trimester at enrolment, bed-net use). These summaries were also computed for the clinical AE occurrence and concomitant medication counts. The concomitant medication was also summarized based frequency of the specific patients and the number of patients who took the medication. In order to aid visualization of the trend of the data, we plotted box plots for the two continuous longitudinal clinical laboratory data (total bilirubin and alanine aminotransferase) outcomes by study visit day.

We considered two different models to compare how they efficiently estimated effect of treatment on clinical AEs over the follow-up time. The models compared were conventional Poisson model M1 and joint model of M9 formulation above. Both the models adjusted for the same set of baseline covariates (maternal age, gravidity and trimester at enrolment). The traditional Poisson model was the first model to be fitted and we used logarithm of the total follow-up time for each patient as an offset.

Then we fitted a joint model with a mixture of expected current value of outcome and random effects association structure as shown in M9. We considered the joint model to assess the joint evolution (/association) between the clinical AEs and other three outcomes (alanine aminotransferase, total bilirubin and concomitant medication) where *α*_1_, *α*_2_, *α*_3_ as defined in M9 are the parameters quantifying the between-outcome association. Secondly, we were also interested investigate the efficiency of the joint model in improving AE incidence rate estimate of the treatment effect obtained from the conventional Poisson model M1. As part of sensitivity analysis we also investigated how the association structure of the joint model affects the magnitude of the treatment effect estimates. Comparing the association structures was achieved by fitting a joint model with random effects association structure only as specified in model M7 above and joint model with mixture of current value and random effects association structure of joint model M9. For both joint models, in order to flexibly model nonlinear changes of the continuous longitudinal clinical laboratory outcomes we used restricted cubic splines with 3 degrees of freedom considered sufficient to enhance flexibility of the model. We compared the fit of the two joint models (M9 versus M7) using the Akaike Information Criterion (AIC), with small values of AIC suggesting better model.

Additionally, we did an exploratory analysis to identify how the frequently reported concomitant medications are associated with the AEs. Such exploratory analysis was helpful in understanding how specific frequently used concomitant medication influence the overall impact of concomitant medication on AEs. Concomitant medication was defined as frequent if it constituted at least 10% of the reported medications. A joint model of a similar structure to M9 but where the concomitant medication count was confined to the specific frequently reported concomitant medication (i.e. paracetamol) was used during the exploratory analysis.

## Results

The trial recruited 870 women who were randomly assigned to received AL, ASAQ, DHAPQ; each arm recruited 290 women. Baseline characteristics were similar across and between the trial arms (Table 1). Maternal age was skewed to the right. Overall, median maternal age was 20 years (IQR: 18.0-24.0). Overall mean haemoglobin concentration at enrolment (g/dl) was 10.1 (SD: 1.3) and distribution was also consistently similar across all the treatment arms. The overall reported bed net use in a night before enrolment was 23.2% such and it was similar across and between the study arms (Table 1).

**Table 1:** Baseline characteristics for women enrolled in PREGACT trial in Malawi

Characteristic	Overall	AL	ASAQ	DHAPQ
Maternal age (years), median (IQR)	20.0 (18.0-24.0)	20.0 (18.0-24.0)	20.0 (18.0-25.0)	20.0 (19.5-24.0)
Gestation age (weeks), median (IQR)	21.0 (18.0-25.0)	21.0 (18.0-25.0)	21.0(18.0-25.0)	21.0 (18.0-24.0)
Haemoglobin(g/dl), mean (SD)	10.1 (1.3)	10.2 (1.4)	10.0 (1.3)	10.1 (1.3)
Primigravida, n (%)	444 (51.1)	144 (49.8)	144 (49.7)	156 (53.8)
Trimester				
Second, n (%)	730 (83.9)	242 (83.4)	244 (84.1)	244 (84.1)
Third, n (%)	140 (16.1)	48 (16.6)	46 (15.9)	46 (15.9)
Bed net use, n (%)	202 (23.2)	65 (22.4)	67 (23.1)	70 (24.1)

In total 1512 AEs were observed over the whole follow-up time and 108 of these were definitely not related to the treatment. Proportion of women who experienced at least one AE in the AL, ASAQ and DHAPQ treatment arms were 64.5% (187 of 300 women), 70.7% (205 of 290 women) and 59.3% (172 of 290 women), respectively. Almost all patients took at least a concomitant medication over the follow-up such that for each arm 99% (287 out of 290) took at least a concomitant medication (Table 2). Across all the treatment arms, the reported median concomitant medications taken was 3; for AL 3(IQR: 2-5), for ASAQ 3(IQR: 1-4) and for DHAPQ 3(IQR: 2-5).

**Table 2:** Summary of AE occurrence and concomitant medication use for women enrolled in PREGACT trial

Characteristic	AL	ASAQ	DHAPQ
Total AEs	475	569	468
AE drug-relatedness totals			
Definitely not	36	34	38
Possibly/probably	26	96	26
Unlikely	413	443	404
At least one AE, n (%)	187 (64.5)	205 (70.7)	172 (59.3)
Total concomitant medications	1229	1033	1042
Median concomitant medications (IQR)	3 (2, 5)	3 (1, 4)	3 (2, 5)
At least one concomitant medication, n(%)	287(99.0)	287(99.0)	287(99.0)

We observed that iron supplement (26.3%), paracetamol (26.2%) and albendazole (10.7%) were frequently reported concomitant medications (Table 3). Almost all the patients analysed, 99.2%, took the iron supplements and approximately two-thirds, 60.3%, of the patients took paracetamol apart from the antimalarial drug under investigation. During the follow-up period, a total of 353 patients reported having used albendazole once.

**Table 3:** Summary of concomitant medication reported by pregnant women in PREGACT trial

Concomitant medication	Number of times reported^b^, n(%)	Number of patients^a^, n(%)
Iron supplement	869 (26.3)	856 (99.2)
Paracetamol	864 (26.2)	520 (60.3)
Others	433 (13.1)	302 (35.0)
Albendazole	353 (10.7)	353 (40.9)
Amoxicillin	220 (6.7)	192 (22.3)
Benzathine	108 (3.3)	68 (7.9)
Pethidine	49 (1.5)	49 (5.7)
Chloramphenicol	48 (1.5)	43 (5.0)
Oral Rehydration Salts (ORS)	47 (1.4)	44 (5.1)
Dextrose	47 (1.4)	45 (5.2)
Lignocaine	39 (1.2)	39 (4.5)
Metronidazole	38 (1.2)	36 (4.3)
Quinine	35 (1.1)	26 (3.0)
Gentamycin	35 (1.1)	35 (4.1)
Sulfadoxine-Pyrimethamine	31 (0.9)	27 (3.1)
Aspirin	16 (0.5)	14 (1.6)
Pitocin	16 (0.5)	16 (1.9)
Erythromycin	13 (0.4)	12 (1.4)
Piriton	12 (0.4)	12 (1.4)
Methyldopa	12 (0.4)	9 (1.0)
Cotrimoxazole	8 (0.2)	8 (0.9)
Promethazine	7 (0.2)	7 (0.8)
Multivitamin Tabs	4 (0.1)	3 (0.4)

^a^This is out of 863 patients who used concomitant medication; some patients took multiple concomitant medications

^b^This is out of 3304 times of reported concomitant medication use

**Fig. 1:**
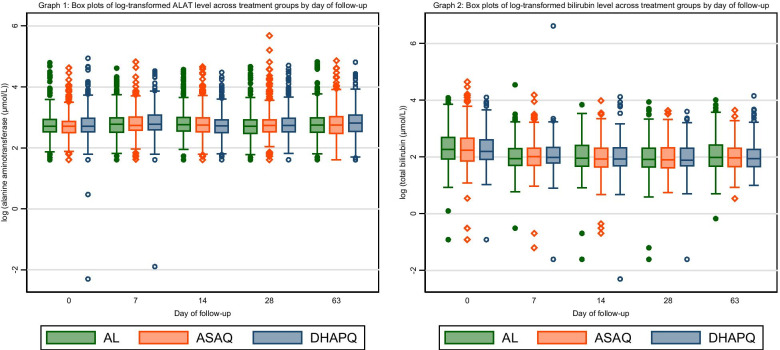
Box plots profiling clinical laboratory safety outcomes for antimalarial drugs over 63-day follow-up among pregnant women in Malawi

Overall median of log-transformed alanine aminotransferase was 2.7 (IQR: 2.5-3.0) such that the medians for AL was 2.8 (IQR: 2.5-3.0), for ASAQ was 2.7 (IQR: 2.5-3.0) and for DHAPQ was 2.8 (IQR: 2.5-3.0). Overall median for log-transformed total bilirubin was 2.0 (IQR: 1.7-2.4). Figure 1 below shows the detailed distribution of each of clinical laboratory safety outcomes over the follow-up days by antimalarial drug treatment arm. As shown in graph 1 within Fig. 1, the alanine amino-transferase did not vary much throughout the follow-up days. The general trend indicated that across the follow-up days, the alanine amino-transferase concentration was slightly higher among the women who received DHAPQ compared to those women who received either AL or ASAQ. After day 0 the total bilirubin concentration gradually decreased across all the treatment arms and, on average, women treated with AL had the highest bilirubin concentration across the follow-up days (see Graph 2 within Fig. 1).

### Parameter estimates for the models

In this section, we report the estimates from the conventional Poisson model (where AEs count is an outcome) (Table 4) and the proposed joint model (Table 5). Our interest was to establish whether the joint model yielded better results than the conventional Poisson model and how the jointly modelled outcomes evolved over the follow-up time in relation to clinical AEs; the results from the AE count sub-model in the joint model (Table 5) are of main interest in our reporting.

**Table 4:** Parameter estimates for clinical AE count incidence using conventional Poisson model

**Characteristic**	**Coefficient**	**SE**	**95% CI**	***P*-value**
Treatment				
*ASAQ*	0.1838	0.0645	0.0574, 0.3102	0.004
*DHAPQ*	–0.0255	0.0679	−0.1587, 0.1076	0.707
Maternal age	0.0208	0.0071	0.0068, 0.0347	0.004
Primigravid	0.1208	0.0731	−0.0224, 0.2641	0.098
Second trimester	0.3768	0.0829	0.1943, 0.5192	< 0.001
Constant	−0.3901	0.2021	−0.7862, 0.0060	0.054

**Table 5:** Parameter estimates from the joint multivariate longitudinal clinical laboratory, concomitant medication and AE counts model

**Variable**	**Coefficient (SE)**	**95% Confidence interval**	***P*-value**
***Log alanine amino transferase longitudinal sub-model***			
Maternal age	0.0006 (0.0023)	− 0.00394, 0.0052	0.793
Treatment			
*ASAQ*	− 0.0036 (0.0275)	−0.0575, 0.0504	0.897
*DHAPQ*	0.0031 (0.0275)	−0.0508, 0.0570	0.909
Second trimester	−0.0212 (0.0311)	− 0.0820, 0.0397	0.496
Constant	2.8221 (0.0609)	2.7027, 2.9415	< 0.001
***Log total*** *** bilirubin longitudinal sub-model***			
Maternal age	−0.0026 (0.0035)	−0.0095, 0.0043	0.464
Treatment			
*ASAQ*	−0.0229 (0.0424)	−0.1059, 0.0601	0.589
*DHAPQ*	−0.0413 (0.0422)	−0.1240, 0.0414	0.328
Second trimester	−0.1786 (0.0493)	−0.2752, − 0.0820	< 0.001
Constant	2.3103 (0.0963)	2.1216, 2.4990	< 0.001
***Concomitant medication count sub-model***			
Maternal age	0.0086 (0.0052)	−0.0017, 0.0189	0.101
Treatment			
*ASAQ*	−0.1841 (0.0619)	−0.3054, − 0.0629	0.003
*DHAPQ*	−0.1723 (0.0610)	− 0.2918, − 0.0528	0.005
Second trimester	0.2861 (0.0718)	0.1454, 0.4268	< 0.001
Constant	0.9147 (0.1395)	0.6414, 1.188	< 0.001
***Adverse events count sub-model***			
Maternal age	0.0189 (0.0088)	0.0017, 0.0362	0.031
Treatment			
*ASAQ*	0.2118 (0.1039)	0.0082, 0.4154	0.041
*DHAPQ*	−0.0508 (0.1046)	−0.2559, 0.1542	0.627
Second trimester	0.3986 (0.1222)	0.1592, 0.6381	0.001
*α*_log(*ALT*)_	− 0.0288 (0.2427)	− 0.5045, 0.4469	0.906
*α*_log(*bilirubin*)_	0.1153 (0.1042)	− 0.0889, 0.3194	0.269
*α*_*concmed*_	1.7487 (0.1029)	1.5471, 1.9503	< 0.001
Constant	−0.8220 (0.7739)	−2.3388, 0.6949	0.288
***Patient-specific random effects standard deviations***			
Standard deviations			
*δ*_log(*ALT*)_	0.2547 (0.0119)	0.2324, 0.2792	
*δ*_log(*bilirubin*)_	0.5025 (0.0154)	0.4732, 0.5336	
*δ*_log(*concmed*)_	0.5378 (0.0248)	0.4914, 0.5887	

*ALT* Alanine aminotransferase, *concmed* Concomitant medication

### Parameter estimates from the conventional Poisson model for the clinical AE count

Using the conventional Poisson model, M9, the clinical AE incidence rate did not differ significantly across the treatment groups (*p* < 0.149). However, the estimates suggested that the AE incidence rate was higher in the ASAQ treatment group compared to the AL treatment group (log IRR: 0.1838; 95% CI: 0.0574, 0.3102; *p* = 0.004) (Table 4). Exponentiating the point estimate of the log IRR yields IRR of 1.2018, implying that the patients treated with ASAQ were expected to have 20.18% higher rate of clinical AEs occurrence compared to those treated with AL. The AE incidence rate did not differ between DHAPQ and AL treatment groups (log IRR: -0.0255; 95% CI: − 0.1587, 0.1076; *p* = 0.707). A year increase in maternal age was associated with 0.0208 increase in log IRR of the AEs (95% CI: 0.0068, 0.0347; *p* = 0.004). The AE incidence rate was significantly higher among the those who enrolled in the second trimester compared to those who enrolled in the third trimester (log IRR: 0.3768; 95% CI: 0.1943, 0.5192; *p* < 0.001).

### Parameter estimates from the joint model

We report the fixed and random parameter estimates for the final fitted joint model based on mixed association structure, M9, (i.e. random effects and current value association structure) in Table 5. AE count sub-model indicated that the AE incidence rate did not vary across all the treatment groups (*p* = 0.376). However, the estimates indicated that AE incidence rate was higher in ASAQ treatment group compared to AL (log IRR: 0.2118, 95% CI: 0.0082, 0.4154; *P* = 0.041) suggesting that patients treated with ASAQ were expected to have 23.59% higher rate of clinical AEs occurrence compared to those treated with AL (i.e. IRR = exp.(0.2118) = 1.2359). The AE incidence rate did not significantly differ in DHAPQ treatment compared to AL (log IRR: -0.0508; 95% CI: − 0.2559, 0.1542). Our results show that the magnitude of the treatment effect was higher in the joint model, M9, compared to the estimates in the conventional Poisson model, M1, above. Among the association parameters describing joint evolution between the outcomes, only concomitant medication was associated with clinical AEs. The association parameter, ***α***_**3**_, linking concomitant medication and clinical AEs suggested that the clinical AEs outcome was positively and strongly associated with the concomitant medications (log IRR: 1.7487; 95% CI: 1.5471, 1.9503; p < 0.001). A unit increase for a patient in deviation from the mean random intercept of concomitant medication use was associated with 5-fold increase in rate of clinical AEs (i.e.IRR = exp.(1.7487) = 5.7471). We found that the clinical AEs were not associated the alanine aminotransferase nor total bilirubin over the follow-up time as demonstrated by their respective association parameters that were non-significant. For the association between alanine aminotransferase and clinical AEs over follow-up time the estimated log IRR was − 0.0288 (95% CI: − 0.5045, 0.4469; *p* = 0.906) and for the association between total bilirubin and clinical AEs the log IRR was 0.1153 (95%CI: − 0.0889, 0.3194; *p* = 0.269). As also observed in the conventional Poisson model above, the clinical AE sub-model indicated that those women who enrolled in the second trimester had a higher AE incidence rate compared to those who enrolled in the third trimester (log IRR 0.3986; 95% CI: 0.1592, 0.6381; *p* = 0.001); the women who enrolled in the second trimester had 48.97% higher rate of clinical AEs (i.e. IRR = exp.(0.3986) = 1.4897) compared to those who enrolled in the third trimester.

In the sub-model for log alanine aminotransferase, we observed no significant difference across all the variables included in that sub-model. For the log total bilirubin sub-model, we observed 0.1786 lower log total bilirubin among the women who enrolled in the second trimester compared to those who enrolled in the third trimester (log IRR: -0.1786; 95% CI: − 0.2752, − 0.0820, *p* < 0.001). The concomitant sub-model showed that concomitant medication was associated with the treatment group. Women enrolled in ASAQ treatment group reported lower incidence rate of concomitant medication use compared to those in AL treatment group (log IRR: -0.1841; 95% CI: − 0.3054, − 0.0629; *p* = 0.003). Women in DHAPQ treatment group also reported lower incidence rate of concomitant medication use compared to those in AL treatment group. Those women who enrolled in the second trimester reported a higher incidence rate of concomitant medication use than those who enrolled in the trial while in the third trimester (log IRR 0.2861; 95% CI: 0.1454, 0.4268; *p* < 0.001).

The standard deviations for patient-specific random intercepts, across the outcomes, suggested that concomitant medication had the highest unobserved heterogeneity with standard deviation of 0.5378 (95% CI: 0.4914, 0.5887). The lowest unobserved heterogeneity was observed in log alanine aminotransferase outcome with random intercept standard deviation of 0.2547 (0.2324, 0.2792).

### Sensitivity analyses

As part of sensitivity analysis, we attempted to relax the normality assumption of the random effects. We fitted a robust joint model for the mixture association structure, M9, where the random effects were assumed to have a t-distribution. The model yielded similar results as those in Table 5 and Alkaike Information Criterion (AIC) of 18,230.28 which was comparable to an AIC of 18,278.53 for the model in Table 5. Secondly, fitting a joint model with random effects association structure, M5, we obtained similar estimates as those in Table 5 and with negligibly lower AIC of 18,277.87.

Following a reviewer’s suggestion, we further investigated the effect of the concomitant medications with high frequencies on AEs. Although iron supplement was the most commonly reported concomitant medication, we did not consider it in assessing the impact of specific concomitant medication on AEs since it was taken by almost all the patients, 99.2%. Instead, we considered paracetamol since it was the second-highest reported concomitant medication where 60.3% of the patients reported it at least once. Assessment of the effect of treatment on clinical AEs using a joint model of a similar structure to M9 but where the concomitant medication count was confined to paracetamol yielded similar results as those obtained in Table 5 (see Table 6). The paracetamol concomitant medication was positively and strongly associated with clinical AEs (log IRR: 1.2969; 95% CI: 1.1328, 1.4609; p < 0.001). However, this observed effect of paracetamol concomitant medication was slightly lower compared to the overall concomitant medication effect as reported in Table 4 (log IRR: 1.7487; 95% CI: 1.5471, 1.9503; p < 0.001). No interaction was observed between the paracetamol concomitant medication and the treatment (*p* = 0.078) such that we focussed on reporting results with the main effect only model as shown in Table 6. The model with interaction terms had a negligibly lower AIC of 16,556.19 than the model without the interaction terms with an AIC of 16,557.29.

**Table 6:** Parameter estimates from the joint multivariate continuous longitudinal clinical laboratory, paracetamol concomitant medication count and AE count model

**Variable**	**Coefficient (SE)**	**95% Confidence interval**	***P*-value**
***Log alanine amino transferase longitudinal sub-model***			
Maternal age	0.0006 (0.0023)	−0.0040, 0.0051	0.810
Treatment			
*ASAQ*	−0.0016 (0.0276)	−0.0557, 0.0525	0.953
*DHAPQ*	0.0031 (0.0275)	−0.0508, 0.0570	0.909
Second trimester	−0.0223 (0.0311)	−0.0833, 0.0387	0.474
Constant	2.8236 (0.0608)	2.7043, 2.9428	< 0.001
***Log total*** *** bilirubin longitudinal sub-model***			
Maternal age	−0.0027 (0.0034)	−0.0094, 0.0039	0.418
Treatment			
*ASAQ*	−0.0346 (0.0419)	−0.1168, 0.0476	0.409
*DHAPQ*	−0.0483 (0.0416)	−0.1299, 0.0334	0.246
Second trimester	−0.1747 (0.0471)	−0.2669, − 0.0824	< 0.001
Constant	2.3118 (0.0911)	2.1394, 2.4966	< 0.001
***Concomitant medication count sub-model***			
Maternal age	0.0144 (0.0088)	−0.0030, 0.0318	0.103
Treatment			
*ASAQ*	−0.3469 (0.1068)	−0. 5563, − 0. 1376	0.001
*DHAPQ*	−0.2850 (0.1046)	− 0.4901, − 0.0798	0.006
Second trimester	0.3850 (0.1273)	0.1355, 0.6344	0.002
Constant	−0.6712 (0.2385)	−1.1387, − 0.2038	0.005
***Adverse events count sub-model***			
Maternal age	0.0161 (0.0092)	0.0019, 0.0340	0.079
Treatment			
*ASAQ*	0.1687 (0.1099)	0.0466, 0.3840	0.125
*DHAPQ*	−0.0876 (0.1103)	− 0.3038, 0.1286	0.427
Second trimester	0.3986 (0.1270)	0.1497, 0.6474	0.002
*α*_log(*ALT*)_	0.0016 (0.2392)	−0.4673, 0.4704	0.995
*α*_log(*bilirubin*)_	0.0218 (0.1207)	−0.2148, 0.2585	0.856
*α*_*concmed*_	1.2969 (0.0837)	1.1328, 1.4609	< 0.001
Constant	−0.6805 (0.7793)	−2.2079, 0.8469	0.383
***Patient-specific random effects standard deviations***			
Standard deviations			
*δ*_log(*ALT*)_	0.2555 (0.0119)	0.2331, 0.2800	
*δ*_log(*bilirubin*)_	0.5028 (0.0153)	0.4736, 0.5338	
*δ*_log(*concmed*)_	0.7931 (0.0480)	0.7043, 0.8931	

*ALT* Alanine aminotransferase, *concmed* Paracetamol concomitant medication

The interest and structure of the proposed joint model could not permit us to assess the impact of albendazole on AEs. The patients who took albendazole took it once; therefore, this concomitant medication arose as a binary outcome that does not meet the joint model specification (i.e. where the concomitant medication is incorporated in the model as a count outcome). Since the objective of the exploratory analysis was to establish the impact of specific frequent concomitant medication on AEs, we did not undertake any additional analysis on the effect of the “other” concomitant medication on AEs.

## Discussion

In clinical trials, multiple safety outcomes collected over the follow-up time require advanced analyses in order to develop a valid drug safety profile that makes full use of the information from the multiple safety outcomes. This paper introduced a framework for joint modelling of multivariate continuous longitudinal clinical laboratory safety outcomes, concomitant medication counts and adverse event count, exploiting the advantages of the existing extended multivariate generalised linear and non-linear mixed effects models framework [23]. Our work focussed on investigating whether clinical AEs varied by treatment arm and how the laboratory outcomes and concomitant medication were associated with clinical AEs over follow-up time in artemisinin-based treatment of malaria during pregnancy clinical trial. We found that the concomitant medication was strongly associated with the clinical AEs incidence, suggesting that the patients who experienced more AEs were more likely to report the use of more concomitant medications. If not well-accounted for such increased use of concomitant medication can distort the treatment effect on the clinical AE occurrence. From methodological perspective, we discovered that the conventional Poisson model underestimated the treatment effect on the AE incidence rate ratio; the effect of treatment on AE incidence rate estimates for the joint model were higher than those from conventional Poisson model. The difference in the models can be attributed to the fact that the joint model uses more of the available information through the explicit modelling of the observed correlation, unobserved heterogeneity and any nonlinear effects [25–27]. The joint model efficiently uses the available information, including the unobserved heterogeneity, leading to more improved estimates compared to the conventional Poisson model involving separate analysis.

Controlling for baseline covariate to address any potential confounding in AE incidence rate ratio estimates, we found that maternal age and enrolling in the second trimester were factors associated with the AE occurrence. A year increase in maternal age was associated with 2% increased rate of AE incidence. This could be as a result that as the women become older they are more likely to report AEs out of experience than the younger women. As expected women who enrolled in the second trimesters had 49% increased rate of AE incidence compared to those who enrolled in the third trimester. This could probably be due to the fact that physiological changes that take place during pregnancy are more impactful in the second than in the third trimester. Hence, the susceptibility to AE occurrence after taking either the antimalarial drug or the concomitant medication (that was strongly associated with increase rate of AE occurrence) since the pregnancy related body physiological changes can interact with the drug.

In assessment of the impact of concomitant medication on AEs, identifying specific concomitant medication is usually of interest. In this paper, we observed that paracetamol greatly contributed to the overall association between concomitant medication and AEs. Although the difference in the effect of overall concomitant medication on AEs and the effect of paracetamol concomitant medication on AEs was not big, the difference could not be considered clinically negligible. Therefore, undertaking both analyses (i.e. effect of overall concomitant medication and effect of specific most frequent concomitant medication on AEs) should be considered useful in understanding antimalarial drug safety profile. Alternatively, an interesting future joint model development can consider quantifying the contribution of each of the specific frequent concomitant medication on AEs occurrence. This can be helpful in identifying and handling any potential noisy concomitant medications that contribute less to the AEs occurrence.

Within the joint modelling framework, our proposed model provides additional knowledge by extending the number of outcomes that can be analysed simultaneously, including multiple longitudinal outcomes and multiple count outcomes, focussing on drug safety assessment. We acknowledge that other researchers have previously proposed similar kind of modelling but applied in the efficacy context [28–32]. Buu et al. considered a joint model of count and binary outcome [30]. Li et al. considered jointly modelling proportion, count and continuous longitudinal outcomes [31]. Yang and Kang introduced a joint model for mixed Poisson outcome and continuous outcome [32]. Our model is considered as a special case of these previously proposed models focussing on drug safety assessment. Key unique features of our proposed joint model include incorporating mixed association structure of random effects and expected value for linking the outcomes, use of restricted cubic splines to accommodate the capturing of any potential time-dependent effects, accommodating multiple safety outcomes and novel application to drug safety assessment. A case study based paper like ours can further facilitate the adoption of improved statistical analysis of safety data in clinical trials [33].

In this paper, data missingness mechanism was ignorable. Our future work will consider investigating the impact of missing data on the model performance under varying follow-up times and model association structures. Secondly, in this paper we worked with a large sample size. An interesting further methodological work would be to assess how the sample size impacts the performance of the model that we applied. Unfortunately, the key limitation of the joint model introduced and applied in this paper is that it is computationally demanding such that it takes longer time to converge. This directly affects the scope of the simulations that can be done to investigate the highlighted potential methodological issues associated with such complicated models. An extension can also be made to the number of outcomes included the joint model depending on the availability of the data. For example, aspartate amino transferase, highly correlated with alanine amino transference, can be added in our joint model.

## Conclusion

Jointly modelling of multivariate longitudinal clinical laboratory safety data, concomitant medication and clinical AEs efficiently harnesses the safety data in order to better understand drug safety profile. This paper has demonstrated the utility of joint modelling to yield improved AE incidence rate estimates compared to the conventional Poisson model. The proposed joint model helps to quantify the association between the laboratory safety outcomes, concomitant medication, and clinical AEs over follow-up time. Key public health relevance of the joint modelling of the safety outcomes includes facilitating the choice of the most important clinical laboratory safety and other safety outcomes for profiling drug safety, especially in resource-constrained settings. In this exploratory analysis, we have also established that the AEs that were observed in the PREGACT trial in Malawi were more linked to concomitant medications that the patients took. Our findings provide an assurance that artemisinin-based treatments can be safely used in second and third trimester during pregnancy.

## Data Availability

The data that support the findings of this study are available from Institute of Tropical Medicine, Antwerp, Belgium but restrictions apply to the availability of these data, which were used under license for the current study, and so are not publicly available. Data are however available from the corresponding author upon reasonable request and with permission of Institute of Tropical Medicine, Antwerp, Belgium (http://www.itg.be).

## Abbreviations

AEs: Adverse events
AL: Artemether–lumefantrine
ASAQ: Amodiaquine–artesunate
DHAPQ: Dihydroartemisinin–piperaquine
IRR: Incidence rate ratio
CI: Confidence interval
PCR: Polymerase-chain reaction
AIC: Akaike Information Criterion
